# Impact of mycoplasma pneumonia infection on urticaria: A nationwide, population-based retrospective cohort study in Taiwan

**DOI:** 10.1371/journal.pone.0226759

**Published:** 2019-12-20

**Authors:** Su-Boon Yong, Wei-Chu Yeh, Hsing-Ju Wu, Huang-Hsi Chen, Jing-Yang Huang, Tung-Ming Chang, James Cheng-Chung Wei

**Affiliations:** 1 Institute of Medicine, Chung Shan Medical University, Taichung, Taiwan; 2 Division of Pediatric Allergy, Immunology and Rheumatology, Department of Pediatrics, Show Chwan Memorial Hospital, Changhua, Taiwan; 3 Department of Nursing, Meiho University, Pingtung, Taiwan; 4 Department of Emergency, Show Chwan Memorial Hospital, Changhua, Taiwan; 5 Research Assistant Center, Show Chwan Memorial Hospital, Changhua, Taiwan; 6 Department of Medical Research, Chang Bing Show Chwan Memorial Hospital, Lukang Town, Changhua County, Taiwan; 7 Department of Medical Research, Chung Shan Medical University Hospital, Taichung, Taiwan; 8 Graduate Institute of Medicine, Kaohsiung Medical University, Kaohsiung, Taiwan; 9 Division of Allergy, Immunology and Rheumatology, Chung Shan Medical University Hospital, Taichung, Taiwan; 10 Graduate Institute of Integrated Medicine, China Medical University, Taichung, Taiwan; Northwestern University Feinberg School of Medicine Galter Health Sciences Library, UNITED STATES

## Abstract

Mycoplasma pneumonia (MP) infection might be pathogenically closely related to urticaria. This study is a nationwide population-based cohort study from 1997 to 2013, which investigated the association between MP infection and urticaria in Taiwan. A total of 1,175 patients were included for the study group, and 2,350 for the control group. Multivariate Cox regression analysis was performed to estimate the adjusted hazard ratio (aHR) for urticaria. Result showed that 254 patients with new-onset urticaria were involved in the study group and 465 incident cases in the control group. The incidence rates (per 100,000 person-months) of urticaria were 37.2 and 32.5 in the study and control groups, respectively. The relative risk is 1.1 (95% CI = 1.0–1.3) indicating no significant correlation between MP and urticaria. The multivariate analysis revealed that the risk of urticaria with MP infection (aHR = 1.1, P = 0.1058) had no statistically significance difference compared to the control group. However, the risk of urticaria in MP-infected patients aged between 20 and 59 years old was found to have increased (aHR = 1.6, 95% CI = 1.1–2.2) prior to a diagnosis.

## Introduction

Urticaria is a vasoactive disorder caused by vasoactive mediators released from activated mast cells which trigger an immune response [1]. The resulted immune response leads to wheals or hives that appear on skin within 6–8 hours and disappear within 48 hours; however, the symptoms can be recurrent several weeks later. Previous study showed that the initiator for urticaria varies largely from food, drugs, chemicals and environmental factors, such as temperature, pressure, as well as animal bites [2–4]. Mechanistically, the initiators induce IgE production, which binds to IgE receptors on mast cells causing histamine release into bloodstream [5].

Albeit urticaria is reportedly non-fatal, it interrupts daily life, such as sleeping due to itching of skin wheals. Clinically, urticaria is categorized into the immunologic and non-immunologic types. Immunologic urticaria is a hypersensitivity reaction mediated by: 1) IgE; 2) IgG autoantibodies; 3) circulating immune complexes to mast cell–expressing Fc receptors for IgG and IgM; and 4) T-cell activation [6]. Non-immunologic urticaria results from mast cell activation through membrane receptors involved in innate immunity, such as complement, toll-like receptor, cytokine, chemokine, opioid or toxicity of xenobiotics (e.g. haptens and drugs) [6]. Furthermore, previous study showed that urticaria is associated with angioedema, a condition of skin swelling in lower dermis and the subcutaneous region that appears as hives. Angioedema is unpredictable, occurring suddenly and lasting up to 72 hours [6, 7]. Nonetheless, the cause of angioedema is not clear.

The association between urticatia and infection has been reported more than 100 years [8, 9]. However, a causal relationship of underlying infection for urticaria is difficult to establish [9]. Evidence suggests that urticaria can be induced by various infections, such as virus and bacterial infections [10], other than food and drugs [11]. Previous studies have focused on viral infection [4, 12, 13], and *Helicobacter pylori* has been reported to be the most bacterial infection associated with urticaria [9], whereas few have focused on mycoplasma pneumonia (MP) infection [14, 15]. Timitilli et al. [16] described MP infection caused unusual clinical manifestations in children, such as urticaria and arthralgia. Furthermore, a Taiwanese study reported that one-third of children with urticaria was related to MP infection with a larger sample size of 114 patients [17]. Recently, children with *Mycoplasma pneumoniae*-related extrapulmonary diseases including urticarial were demonstrated to have significantly higher total serum IgE levels than those with classical respiratory infections by *M*. *pneumoniae* [18]. However, these studies only focused on pediatric patients. On the other hand, the study of Lim et al. [19] demonstrated that acute MP infection in 15 (30.6%) of 49 adults with acute urticaria compared to 3 (6.8%) of 44 adults with chronic urticaria, suggesting that *M*. *pneumoniae* may also play a role in the etiology of acute urticaria in adult. Therefore, this study aims to examine the correlation between MP and urticaria through understanding the underlying mechanism for MP-induced urticaria symptoms.

## Methods

### Data source

This was a retrospective study, because the data collected in this study are from an existing record, the National Health Insurance Research Database (NHIRD) from the Taiwan National Health Insurance (NHI) Program, which is managed by National Health Research Institutes (NHRI) [20, 21]. This retrospective data can be immediately analyzed to determine the relative risk of the study group compared to the control group. Under the NHI Program, 98% of Taiwanese benefit from the compulsory, single-payment health care system since 1996. All data derived from the NHIRD are anonymized for epidemiologic research that contains the information of patients’ demographics, health care service data, medication dispensation, International Classification of Diseases, and so forth. Due to the large sample size provided by the NHIRD, previously reported diagnostic information of the Longitudinal Health Insurance Research Database (LHIRD) derived from the NHIRD has been established in 2000. The data used in this study is de-identified, and is derived from the LHIRD from 1997 to 2013. The de-identified, secondary data required no informed consent, and was approved by the Institutional Review Board of Chung Shan Medical University Hospital (CS15134).

### Exposure to *Mycoplasma pneumoniae*

**Fig 1:**
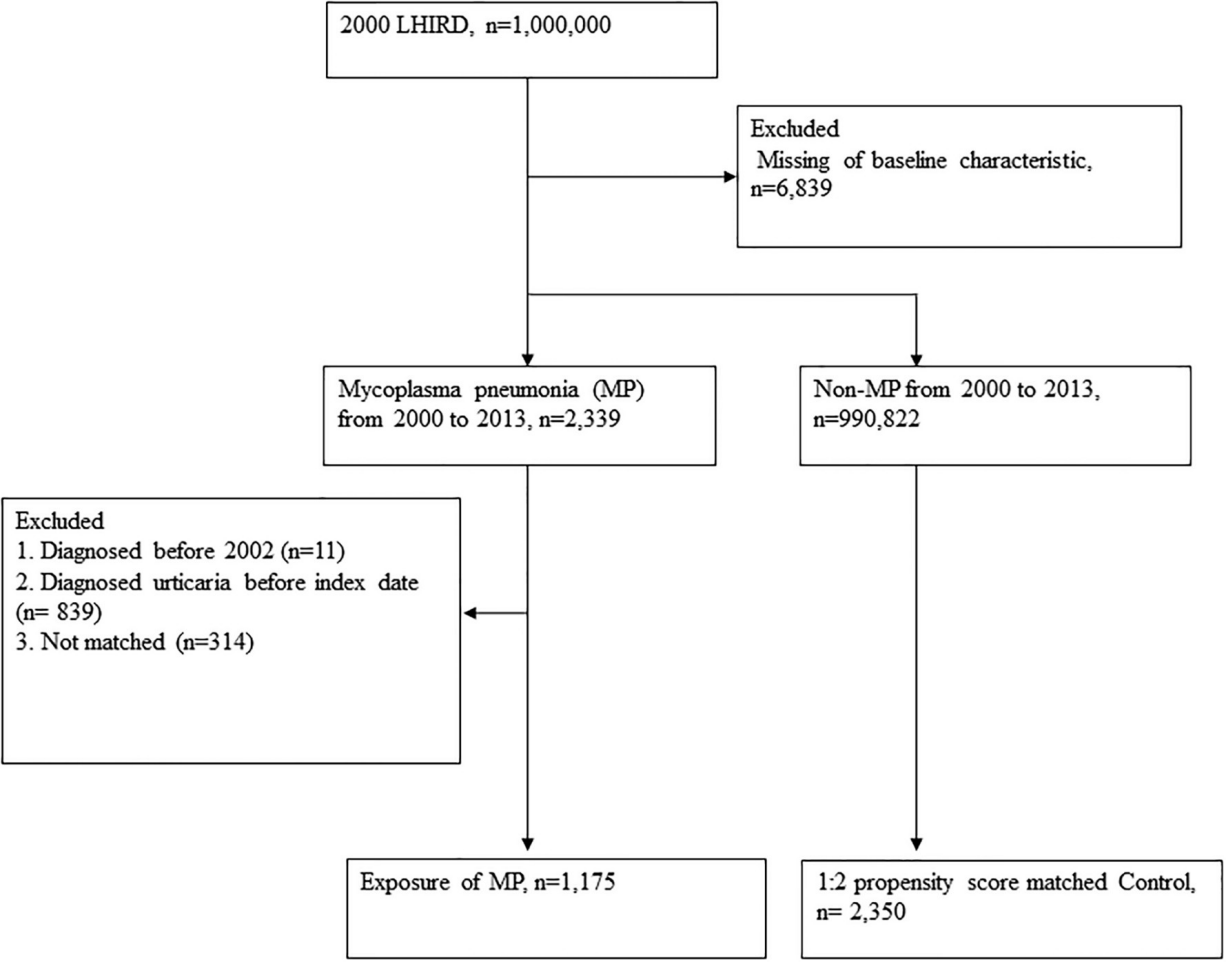
Flowchart depicting the incidence number used in this study for patients with mycoplasma pneumonia infection. LHIRD: Longitudinal Health Insurance Research Database, MP: Mycoplasma pneumonia.

Patients with a first-time diagnosis of MP were identified from the NHIRD by using International Classification of Diseases, Ninth Revision, Clinical Modification (ICD-9-CM) code 483.0 between January 1, 1997 and December 31, 2013. Because the administrative datasets are always criticized for their poor diagnosis validity, this study defines the MP patients who had emergency department (ED) visit or hospitalization for MP in their medical claims. We excluded 11 patients who were diagnosed with urticaria before 2002; and 839 patients who were diagnosed with MP before urticarial diagnosis. In addition, 1,175 patients with MP were included as exposure individuals. We assigned the ED visit for receiving a diagnosis of MP as the index date. The four controls were age and sex, individually matched with one MP patient, and they were still at risk at the index date. Furthermore, each individual in the study group was propensity score-matched with a non-MP individual. Details (including control group) are being discussed in the following sections. Propensity score matching was performed at a ratio of 1:2 to correct potential confounders including age, gender, and comorbidities. Finally, we included 1,175 patients with MP and 2,350 propensity score matched controls in this study (Fig 1).

### Case definition of MP

The identification of patients with urticaria was based on records ascertained by using ICD-9-CM codes 708.x. Patients with urticaria needed to have at least two outpatient visits or one hospital admission to fulfill the case definition. In addition to this, we also find co-morbidities diseases related to urticaria and MP infection, which included rheumatoid arthritis (RA: ICD-9-CM codes 714.0), asthma (ICD-9-CM 493), allergic rhinitis (ICD-9-CM 477), diabetes mellitus (ICD-9-CM 250), zoster (ICD-9-CM053.X, 054.X), hepatitis B virus infection (ICD-9-CM070.2, 070.3, V02.61), hepatitis C infection (ICD-9-CM070.44, 070.51, 070.54, 070.7, V02.62), gout (ICD-9-CM274). These co-morbidities were considered as the covariates in the multivariate analysis.

### Statistical analysis

The absolute standardized difference (aSD) was the indication of difference in study variables between the study and control groups, particularly the population-based and large sample study. The small (<10%) standardized difference agreed with the balance between groups. The Cox proportional hazard regression models were used to calculate the hazard ratios (HR). There were 95% confidence intervals (CI) of associations between pre-existing urticaria and MP infection after adjusting at index date for age, sex, urbanization, co-morbidities or medication usage. The Kaplan-Meier curve was performed for the cumulative probability of urticaria for patients with MP infection. All data analyses were performed using SAS 9.4 (SAS Institute Inc., Carey, NC), P value less than 0.05 is considered to be statistically significant.

## Results

### Study population

The selection of study participants is shown in Fig 1. After we excluded those cases missing demographic data that died before 2002, a total of 2,339 MP-infected patients were identified with at least two outpatient visits or one admission for urticaria between 2000 and 2013. We further excluded those urticaria cases diagnosed before 2002 and MP cases diagnosed before index date. Result showed that 1,175 patients with newly-diagnosed MP infection cases were included.

### Baseline characteristics of patients with MP

Result shows that 49.4% and 50.6% of the MP-infected patients were female and male, respectively. After propensity score matching, the difference of baseline characteristics were all small (10%) among the study groups (Table 1). Table 1 shows that most MP-infected patients (84.5%) were not admitted to hospital before index date in propensity score matching groups. However, age-sex matching groups showed that 67.5% MP-infected patients were not admitted to hospital before the index date. The serious MP-infected cases might have been excluded after propensity score matching.

**Table 1:** Baseline characteristics among study groups.

	1:4 age and sex matching (before PSM)			1:2 (PSM)		
	Control n = 5,940	MP n = 1,485	aSD (%)	Control n = 2,350	MP n = 1,175	aSD (%)
Sex			0.0			42.6
Female	2916(49.1%)	729(49.1%)		1144(48.7%)	580(49.4%)	
Male	3024(50.9%)	756(50.9%)		1206(51.3%)	595(50.6%)	
Age			0.0			3.9
<6	548(9.2%)	137(9.2%)		193(8.2%)	105(8.9%)	
6–11	1964(33.1%)	491(33.1%)		827(35.2%)	402(34.2%)	
12–19	872(14.7%)	218(14.7%)		372(15.8%)	185(15.7%)	
20–39	1100(18.5%)	275(18.5%)		466(19.8%)	233(19.8%)	
40–59	564(9.5%)	141(9.5%)		182(7.7%)	99(8.4%)	
> = 60	892(15.0%)	223(15.0%)		310(13.2%)	151(12.9%)	
Urbanization			3.6			1.7
Urban	3502(59.0%)	856(57.6%)		1352(57.5%)	666(56.7%)	
Sub-urban	1814(30.5%)	478(32.2%)		774(33.0%)	395(33.6%)	
Rural	624(10.5%)	151(10.2%)		224(9.5%)	114(9.7%)	
Low income	24(0.4%)	10(0.7%)	3.7	16(0.7%)	6(0.5%)	2.2
Length of hospital stays			54.1			6.0
No admission	5268(88.7%)	1003(67.5%)		1976(84.1%)	993(84.5%)	
1–6 days	412(6.9%)	229(15.4%)		272(11.6%)	131(11.2%)	
7–13 days	129(2.2%)	102(6.9%)		70(3.00%)	28(2.4%)	
> = 14 days	131(2.2%)	151(10.2%)		32(1.4%)	23(2.00%)	
Co-morbidity						
Asthma	464(7.8%)	301(20.3%)	36.5	304(12.9%)	164(14.0%)	43.0
Allergic rhinitis	1142(19.2%)	468(31.5%)	28.5	662(28.2%)	312(26.6%)	3.6
Zoster	170(2.9%)	56(3.8%)	5.1	87(3.7%)	39(3.3%)	2.1
HBV infection	62(1.0%)	14(0.9%)	1.0	22(0.9%)	11(0.9%)	0.0
HCV infection	19(0.3%)	17(1.1%)	9.7	5(0.2%)	3(03%)	8.8
Diabetes mellitus	302(5.1%)	105(7.1%)	8.3	116(4.9%)	57(4.9%)	0.4
Gout	14(0.2%)	10(0.7%)	6.5	7(0.3%)	3(0.3%)	0.8
Cancer	44(0.7%)	17(1.1%)	4.2	23(1.0%)	14(1.2%)	2.1
Medication						
Corticosteroids	122(2.1%)	73(4.9%)	15.7	60(2.6%)	40(3.4%)	5.0
NSAID	516(8.7%)	235(15.8%)	21.9	255(10.9%)	129(11.0%)	0.4
Antihistamines	1180(19.9%)	472(31.8%)	27.5	609(25.91 = %)	311(26.5%)	1.3

Abbreviations: aSD, absolute standardized difference, the value was presented as (%) = original aSD × 100, and rounded the values to one decimal place; MP: mycoplasma pneumonia; PSM: propensity score match.

### Time to event analysis

**Fig 2:**
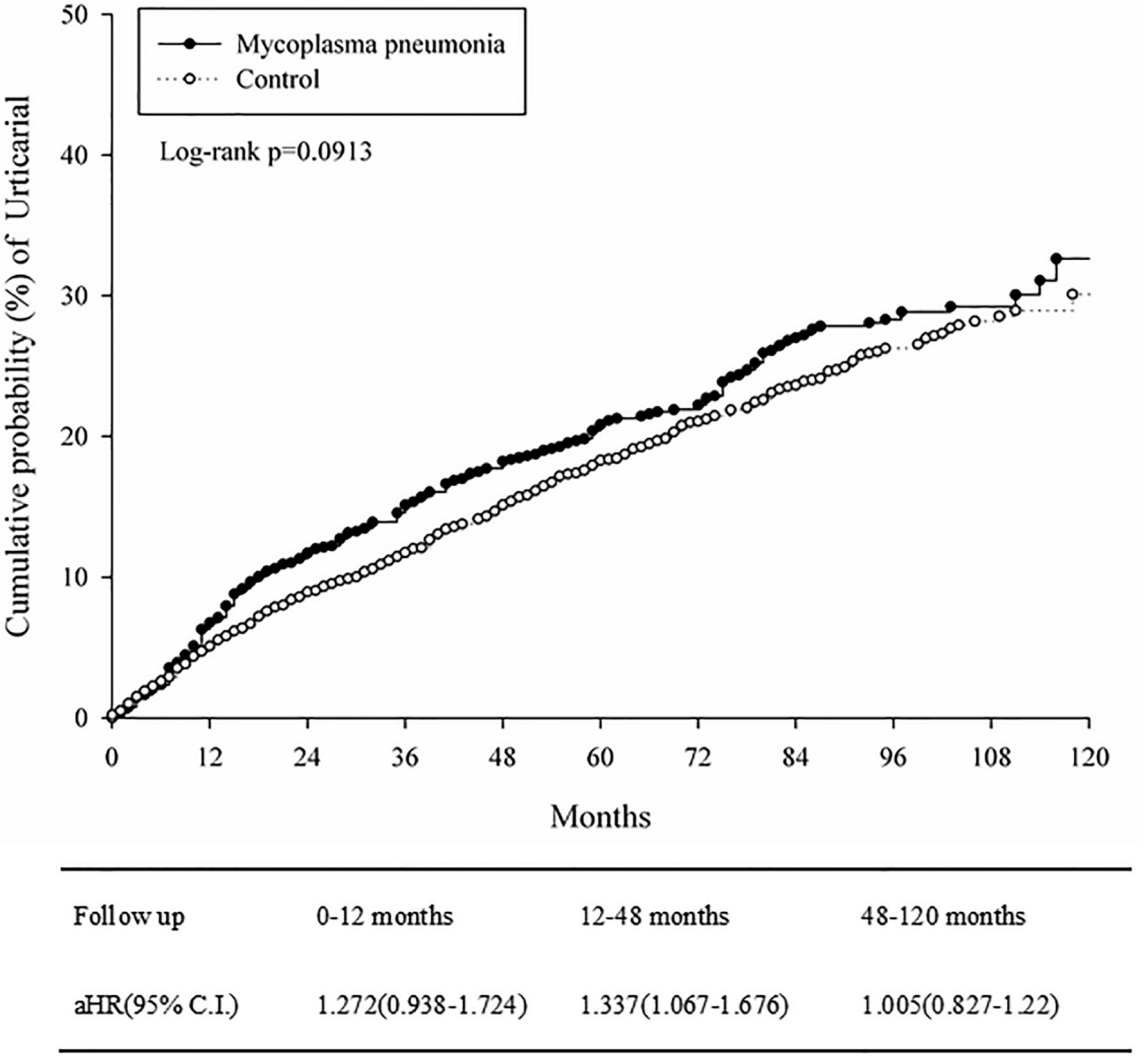
Kaplan-Meier curves of cumulative probability of urticaria in propensity score matching study groups.

Table 2 shows the incidence rates (per 10,000 person a month) of urticaria were 37.2 (95% CI = 32.9–42.1) and 32.5 (95% CI = 29.7–35.6) in MP-infected and control group, respectively. The Kaplan-Meier curves of cumulative probability of urticaria in propensity scored matching study groups were shown in Fig 2, there was a borderline significantly higher incidence risk in the MP-infected group compared to the control group (log rank *p* = 0.0913). The adjusted HR (aHR) at 0–12 months, 12–48 months and 48–120 months were 1.272 (95% CI = 0.938–1.724), 1.337 (95% CI = 1.067–1.676) and 1.005 (95% CI = 0.827–1.22), respectively. The aHR for urticaria among subjects with MP was 1.2 (95% CI = 1.0–1.4; P = 0.0086) after 1:4 age and sex matching (Table 3). However, the aHR for urticarial became non-significant after 1:2 PSM (aHR = 1.1; 95% CI = 1.0–1.3; P = 0.1058, Table 3).

**Table 2:** Incidence of urticaria in study group.

	1:4 age and sex matching		1:2 PSM	
	Control n = 5,940	MP n = 1,485	Control n = 2,350	MP n = 1,175
Follow up person months	363226	84195	142896	68286
New case of urticaria	1053	322	465	254
Incidence rate* (95% CI)	29.0 (27.3–30.8)	38.3 (34.3–42.7)	32.5 (29.7–35.6)	37.2 (32.9–42.1)
Relative risk (95% CI)	Reference	1.3 (1.2–1.5)	Reference	1.1 (1.0–1.3)

* Incidence rate, per 10000 person months

**Table 3:** HR for urticaria.

	1:4 age and sex matching		1:2 PSM	
	Adjusted HR (95% CI)	P value	Adjusted HR (95% CI)	P value
Mycoplasma pneumonia	1.2(1.0–1.4)	0.0086	1.1(1.0–1.3)	0.1058
Sex				
Female	Reference		Reference	
Male	0.8(0.8–0.9)	0.0017	0.8(0.7–0.9)	0.0011
Age				
<6	Reference		Reference	
6–11	1.0(0.9–1.2)	0.5678	1.1(0.8–1.4)	0.6129
12–19	1.0(0.8–1.2)	0.7956	0.9(0.7–1.3)	0.6362
20–39	1.0(0.8–1.3)	0.8758	1.2(0.9–1.6)	0.2436
40–59	0.9(0.6–1.1)	0.2882	1.0(0.7–1.6)	0.8946
> = 60	0.8(0.6–1.1)	0.1206	1.0(0.7–1.5)	0.8964
Urbanization				
Urban	0.9(0.8–1.1)	0.4895	1.1(0.8–1.4)	0.6349
Sub-urban	1.0(0.9–1.3)	0.6607	1.3(1.0–1.8)	0.0778
Rural	Reference		Reference	
Low income	2.1(1.2–3.7)	0.0123	2.4(1.2–4.6)	0.0107
Length of hospital stays (Within 2 year before index date)				
No admission	Reference		Reference	
1–6 days	1.0(0.8–1.2)	0.8784	1.1(0.8–1.3)	0.6600
7–13 days	1.1(0.8–1.5)	0.5221	0.9(0.5–1.7)	0.8386
> = 14 days	0.8(0.5–1.2)	0.2196	1.2(0.4–3.8)	0.7558
Co-morbidity				
Asthma	1.0(0.8–1.2)	0.8817	0.9(0.7–1.1)	0.4529
Allergic rhinitis	1.3(1.1–1.5)	0.0003	1.3(1.1–1.6)	0.0006
Zoster	1.0(0.7–1.4)	0.9471	1.2(0.8–1.8)	0.2857
HBV infection	1.2(0.7–2.3)	0.5111	1.0(0.4–2.7)	0.9916
HCV infection	1.0(0.4–2.8)	0.9734	-	-
Diabetes mellitus	1.0(0.7–1.5)	0.9191	0.7(0.4–1.3)	0.2711
Gout	0.5(0.1–3.7)	0.5127	1.0(0.1–7.7)	0.9661
Cancer	1.3(0.5–3.2)	0.6107	0.6(0.1–2.9)	0.5088
Medication				
Corticosteroids	1.2(0.9–1.7)	0.2210	1.1(0.7–1.8)	0.5795
NSAID	1.1(0.9–1.3)	0.4666	1.0(0.8–1.3)	0.8693
Antihistamines	1.4(1.2–1.6)	< .0001	1.4(1.2–1.7)	0.0004

### Subgroup analysis in propensity score matched groups

Table 4 shows that MP-infected patients at low income group had higher but no significant risk of urticaria with aHR of 3.3 (95% CI = 0.4–28.7) in comparison with the control group. In the group aged 20–59 years old, the incidence of urticaria was significantly higher in MP-infected patients with aHR of 1.6 (95% CI = 1.1–2.2) compared with the control group.

**Table 4:** Subgroup analysis in PSM groups.

	Rate (per 10000 person months)		
Subgroup	In control	In MP exposure	aHR (95% CI)
Sex			
Male	29.2(25.6–33.4)	33.0(27.5–39.7)	1.1(0.9–1.4)
Female	36.0(31.9–40.8)	41.4(35.1–48.8)	1.1(0.9–1.4)
p for interaction			0.9344
Age at baseline			
<20	34.4(30.9–38.2)	35.4(30.5–41.2)	1.0(0.9–1.3)
20–59	28.1(22.9–34.4)	44.3(35.0–56.1)	1.6(1.1–2.2)
> = 60	29.4(20.7–41.8)	31.6(18.7–53.4)	0.8 (0.4–1.7)
p for interaction			0.0775
Low-income			
No	32.4(29.6–35.5)	36.8(32.5–41.6)	1.1(1.0–1.3)
Yes	48.5(20.2–116.4)	156.9(58.9–417.9)	3.3(0.4–28.7)
p for interaction			0.1343
Allergic rhinitis			
No	28.7(25.6–32.2)	33.8(29.0–39.4)	1.18(1.0–1.4)
Yes	41.9(36.1–48.6)	45.7(37.2–56.3)	1.1(0.8–1.4)
p for interaction			0.5359
Antihistamines			
No	28.6(25.5–32.1)	33.7(28.9–39.4)	1.2(1.0–1.4)
Yes	41.9(36.2–48.6)	45.1(36.8–55.1)	1.1(0.8–1.4)
p for interaction			0.5094

## Discussion

This is the first study to apply nationwide longitudinal population-based database to estimate the relationship between MP infection and urticaria whereby MP infection is proposed as a developmental risk factor for urticaria since MP infection has been linked to inflammatory disease, such as arthritis [22]. As far back as 1920s, physician J. Goodwin Tomkinson reported that infection can be the cause of urticaria, followed by H. W. Barber who reported that focal sepsis of *Streptococci* and *Staphylococci* infections can be the causes of chronic or recurrent urticarial [23, 24]. Based on our analysis of the NHIRD in Taiwan from 1997 to 2013, MP infection is associated with urticaria. In this study, we highlight the importance that patients with MP infection ought to be evaluated for urticarial risk.

Patients with MP infection were treated with antibiotics commonly used to treat bacterial infections, azithromycin (10 mg/kg/day) for 3 days. Wu CC et al. found that azithromycin-treated urticaria patients had significantly shorter mean (SD) convalescence period, shorter duration for complete resolution when compared to untreated urticaria patients [17, 23]. This result implies that antibiotic azithromycin effectively affects the duration for urticarial symptoms. Therefore, this study supports the association between *M*. *pneumoniae* and urticaria; when pediatric patients with urticarial are not responding to antihistamine treatment, they should be encouraged to examine for the presence of MP infection [17].

Urticaria is a benign disease for adults and children since the symptoms are often short-lived, and can be treated with antihistamine. Clinically, patients hospitalized for severe urticaria, or having refractory urticaria are difficult to be treated by antihistamines and any other types of treatments. MP infection has been observed and implicated to play a role in the refractory urticaria. MP infection in adults and children can lead to complications in lungs and other organ (e.g. kidney), as well as skin disorders, such as Stevens-Johnson syndrome and erythema multiforme [25]. Interestingly, the known mechanism for MP-associated skin disorder, Stevens-Johnson syndrome, is attributed to cytotoxic T-cell-mediated immune response, cell death mediators, such as FasL and annexin A1 from the apoptotic keratinocytes in skin tissue, not by invading foreign *Mycoplasma* [26]. As a result, patients with refractory urticaria who are unresponsive to antihistamine treatment are encouraged to take evaluation for MP infection. The outcome of the diagnosis can determine the reason for antibiotic-responsive disorder, and reduce the use of corticosteroid, such as cyclosporine [27]. In addition, previous study has implicated that acute urticaria found in children are not IgE-mediated allergic response, but may be related to various infections of the upper respiratory tract [17]. The symptom of urticaria in children that includes febrile episode suggests an infection. Furthermore, evidence shows that 39% to 48% of children having acute urticaria are indeed infected with microbes [13]. Taken together, the cause of urticaria is likely to be associated with the MP infection.

There are several limitations to this study. Firstly, there is the lack of public alertness for MP infection, leading to undiagnosed MP infection and lack of medical service, and thus less people seek urticaria treatment. Secondly, due to the variation in clinical definition for urticaria, potential MP infection can be omitted as a risk factor during diagnosis. Thirdly, severe MP-infected patients who were hospitalized were not included in this study. In addition, the data in this study is limited to Taiwanese population; therefore, other ethnic groups and nations worldwide might not be applied in this finding. However, the data analyzed in this study is pioneer, and is convincing due to the randomized, and large sample size.

## Conclusions

This 13-year population-based cohort epidemiological study identified an increased risk for urticaria among patients with MP infection. This study provided insight into early awareness of MP infection among urticaria patients and early antibiotic treatment taken as precaution for high risk patients.
